# Epitope mapping using immunopeptidomics reveals novel immunodominant CD8 T cell epitopes of the AAV9 capsid

**DOI:** 10.3389/fimmu.2025.1641289

**Published:** 2025-08-08

**Authors:** Akhila Balasubramanian, Marek Prachar, Birgit Klaproth, Victoria Copeland, Sune Justesen, Yi Wen, Robert W. Siegel, Laurent P. Malherbe

**Affiliations:** ^1^ Lilly Research Laboratories, Eli Lilly and Company, Indianapolis, IN, United States; ^2^ Lilly Oncology, Eli Lilly and Company, Copenhagen, Denmark

**Keywords:** AAV9, immunopeptidomics, HLA class I, CD8 T cells, capsid epitopes, immune monitoring

## Abstract

**Introduction:**

Adeno-associated virus (AAV)-mediated gene therapy is a promising approach to treat genetic disorders, offering advantages such as high transduction efficiency, diverse tissue tropism, and an acceptable safety profile. However, the immunogenicity of AAV vectors, particularly the activation of AAV capsid-specific CD8 T cells, significantly impacts therapeutic efficacy and safety. Capsid-specific T cell responses are currently monitored in the clinic using overlapping peptides, which do not represent the naturally presented capsid immunopeptidome. Our previous work identified the naturally presented peptides of AAV capsids using MHC-associated peptide proteomics (MAPPs).

**Methods:**

In this study, we compared overlapping and MAPPs-derived peptides of the AAV9 capsid in their capacity to trigger capsid-specific T cell responses in healthy donor PBMCs.

**Results:**

Both peptide groups induced measurable T cell responses in FluoroSpot assays only after an expansion phase, reflecting the low frequency of circulating capsid-specific T cells in both seropositive and seronegative donors. Surprisingly, overlapping and MAPPs-derived capsid peptides expanded largely distinct T cells that did not cross-react. The T cell response to MAPPs-derived capsid peptides was dominated by capsid-specific CD8 T cells recognizing peptides eluted from HLA Class I, allowing us to identify CD8 T cell capsid epitopes in healthy donors. By screening 13 matrix pools (comprising 41 HLA Class I MAPPs peptides of the AAV9 capsid) in 24 healthy donors using FluoroSpot assays, we identified ten epitopes eliciting IFN-γ release in at least one donor. 9 of the 10 identified epitopes were novel, varied between 9–13 amino acids in length, and displayed strong binding to their predicted HLA binding alleles. Only one of four previously reported capsid CD8 T cell epitopes elicited a response in the tested cohort of healthy donors with diverse HLAs. Remarkably, in two instances where MAPPs peptides of different lengths were presented on HLA Class I, CD8 T cell response was only observed to longer epitopes.

**Discussion:**

Our results represent the first extensive analysis of the naturally presented peptides of the AAV9 capsid. This work provides strategies to improve the detection of the capsid-specific CD8 T cell response in the clinic and reduce vector immunogenicity through the identification of novel CD8 T cell epitopes.

## 1 Introduction

Adeno-associated viruses (AAVs) are among the most widely used delivery vectors for gene therapies. Key attributes such as a low risk of genomic integration, ability to transduce different tissues, and an acceptable immunogenicity profile make these vectors an attractive option for gene delivery in patients (1). Currently, 6 FDA-approved AAV-based gene therapies are available for the treatment of various genetic disorders- Luxturna^®^ (retinal dystrophy), Zolgensma^®^ (spinal muscular atrophy), Hemgenix^®^ (hemophilia B), Roctavian^®^ (hemophilia A), Elevidys^®^ (Duchenne muscular dystrophy), and Kebilidi^®^ (aromatic L-amino acid decarboxylase deficiency) (2, 3).

Despite their proven utility, AAV-based gene therapies are met with a significant immune response in patients, which impacts their efficacy. This immune response can be triggered by several components, including the AAV capsid protein, gene editing enzymes, or the transgene product (4). The anti-capsid immune response, in particular, can have innate, humoral, and cellular components, compounded by pre-existing immunity that develops due to exposure to wildtype AAVs (1, 5). The cellular immune response against the capsid comprises CD8 cytotoxic and CD4 helper T cells that recognize capsid peptides presented on HLA Class I and Class II molecules, respectively (6). The CD8 T cell response is of particular importance as it can directly target transduced cells, leading to loss of transgene expression and efficacy. This has been demonstrated in clinical trials for hemophilia, where the loss of Factor IX gene expression post therapeutic infusion coincided with the detection of capsid-specific CD8 T cells and elevated liver transaminases (7–9). Monitoring capsid-specific T cell response is therefore critical for advancing the safety and efficacy of AAV-based gene therapies.

Capsid-specific T cell responses are typically measured by ELISpots using overlapping 15-mer peptides that span the entire AAV capsid protein. The primary shortcoming of this approach is that these overlapping peptides may not reflect the naturally presented capsid peptides that T cells encounter *in vivo.* MHC-associated peptide proteomics (MAPPs) is a powerful technique for identifying naturally presented capsid peptides on HLA molecules (10, 11) that can be leveraged to identify potential immunodominant epitopes (12). However, how overlapping and MAPPs-derived capsid peptides compared in their abilities to a) detect capsid-specific T cells and b) identify immunodominant capsid epitopes, has not been explored thus far.

Using MAPPs, we previously identified capsid peptides in several AAV serotypes that are naturally presented on HLA Class I and HLA Class II (13, 14). In the current study, we compared overlapping and MAPPs-derived peptides of the AAV9 capsid in their capacity to induce T cell responses and highlight key differences in the capsid-specific T cell responses captured by either group of peptides. We found that the T cell responses to MAPPs-derived peptides were dominated by CD8 T cells that recognized novel immunodominant CD8 T cell epitopes with unique lengths and strong binding to their predicted HLA alleles. These results can help inform strategies for measuring and mitigating the capsid-specific CD8 T cell response in AAV-based gene therapies.

## 2 Materials and methods

### 2.1 Cohort selection, PBMCs, and plasma samples

Peripheral Blood Mononuclear Cells (PBMCs) and plasma samples of consenting healthy donors were purchased from Cellular Technology Limited (CTL). Initial assay development and expansion experiments were performed on a cohort of 20 healthy donors, comprising 10 AAV9 seropositive and 10 AAV9 seronegative individuals (**Supplementary Table 1**). Deconvolution studies to identify immunodominant CD8 T cell epitopes were conducted on a cohort of 24 healthy donors, which included 17 donors from the initial cohort (3 donors were no longer available due to vendor stock exhaustion) as well as 7 additional donors. Additional donors were selected to ensure representation of predicted peptide binding alleles that were absent in the initial cohort. In total, PBMCs and/or plasma from 27 healthy donors were utilized in the study. Donor IDs and HLA allele information for all the donors can be found in **Supplementary Table 2**.

### 2.2 Total antibody assay for the measurement of anti-AAV9 antibodies

Pre-existing anti-AAV9 antibodies were measured in human plasma for a cohort of 76 healthy donors using the affinity capture and elution (ACE) TAb assay as previously published (15). Briefly, Nunc Maxisorp 96-well plates were incubated for 1 hour with 100 µL per well of AAV9-GFP (8E10 DRP/mL) and washed. Human plasma samples and controls were diluted 1:10 in Tris-buffered saline and added to the washed plates, followed by overnight incubation on a shaker at 4 degrees Celsius. The following day, plates were washed and treated with 65 µL per well of 300 mM acetic acid. After a 5-minute incubation, 50 µL of the acid-eluted antibodies were transferred to MesoScale Discovery (MSD) plates containing 50 µL of 1 M Tris-HCl neutralization buffer (pH 9.5). The MSD plates were incubated for 2 hours at room temperature to allow the immobilization of anti-AAV9 antibodies. Following this, the MSD plates were washed, treated with 100 µL per well of ruthenium labeled AAV9-GFP (9.8E10 DRP/mL), and incubated for 1 hour at room temperature. After a final wash, 2X MSD read buffer was added and signals were collected in an MSD Quick Plex SQ 120 reader. Donors with antibodies above the assay Tier 1 cut point were defined as seropositive and those below the assay Tier 1 cut point were defined as seronegative (**Supplementary Table 1**).

### 2.3 Peptide pools for initial expansion studies

The overlapping (O/L) peptide pool contained peptides spanning the VP1 capsid protein of AAV9, organized as 15-mers with a 11 amino acid overlap (182 peptides). The MAPPs-derived peptide pools comprised naturally presented peptides of the VP1 capsid protein of AAV9 identified through MAPPs. Three MAPPs-derived peptide pools were utilized for the study- Class I MAPPs pool (containing 41 MAPPs-derived capsid peptides presented on HLA Class I), Class II MAPPs pool (containing 12 MAPPs-derived capsid peptides presented on HLA Class II), and the total MAPPs pool (containing 53 MAPPs-derived peptides, combining HLA Class I and Class II peptides) (**Supplementary Table 3**). Peptide pools were dissolved in 100% DMSO and tested at 1 µg per peptide/mL. All peptide pools were synthesized by GenScript.

### 2.4 Matrix pools and individual peptides for peptide deconvolution studies

For the identification of immunodominant CD8 T cell epitopes, the 41 HLA Class I MAPPs peptides were arranged in a matrix of 13 pools, each containing 6–7 peptides (**Supplementary Table 4**). Per the matrix, each matrix pool was constructed by combining the peptides listed in each column or each row (16). Peptides present at the intersection of responding matrix pools were shortlisted and tested individually. All matrix pools were dissolved in 100% DMSO and tested at 1 µg per peptide/mL. For individual testing, peptides were dissolved in 100% DMSO and tested at 2 µg/mL. Matrix pools and individual peptides were synthesized by GenScript.

### 2.5 Direct *ex vivo* FluoroSpot assay with frozen PBMCs

The manufacturer’s instructions were followed for FluoroSpot plate set up and processing. AIM V containing 5% Serum Replacement (SR) (Gibco) was used as the culture media. Frozen PBMCs were thawed in the water bath and rested overnight in the CO2 incubator at 37 degrees Celsius. The following day, rested PBMCs were washed and plated in triplicates (250,000 cells/well) on precoated IFN-γ/TNF-α FluoroSpot plates (MabTech) along with test peptide pools (1 µg per peptide/mL). Anti-CD3 antibody (MabTech, 100 ng/mL) and DMSO were used as positive and negative controls respectively. Plates were incubated at 37 degrees Celsius for ~ 24 hours, washed, and treated with primary and secondary antibodies. Processed plates were dried and read on the MabTech IRIS instrument to record the number of spots per well.

### 2.6 FluoroSpot assay with expanded PBMCs

Frozen PBMCs were thawed and resuspended in AIM V+ 5% SR at 1X10^6 cells/mL. Cells were stimulated with test peptide pools (1 µg per peptide/mL) and expanded *in vitro* for 10 days as described previously (17). On Days 4 and 7, cells were supplemented with a cytokine cocktail containing IL-2 (R&D Systems, 10 units/mL), IL-7 (R&D Systems, 10 ng/mL), and IL-15 (PeproTech, 10 ng/mL). On Day 10, cells were harvested, washed, and restimulated on precoated IFN-γ FluoroSpot plates (MabTech) in triplicates (125,000 cells/well). Peptide pools (1 µg per peptide/mL) or individual peptides (2 µg/mL, for deconvolution studies) were used for restimulation. Anti-CD3 antibody (MabTech, 100 ng/mL) and DMSO were used as positive and negative controls respectively. Plates were incubated at 37 degrees Celsius for ~ 24 hours and processed as described in the previous section.

### 2.7 T cell depletion

For experiments involving T cell depletion, CD4 T cells were depleted via magnetic separation using human CD4 Microbeads (Miltenyi Biotec) and an AutoMACS separator (Miltenyi Biotec) according to manufacturer’s instructions. Depleted cells were washed with MACS buffer prior to seeding on culture plates. An average of 85-95% T cell depletion was achieved in all donors as confirmed through flow cytometry.

### 2.8 Peptide/HLA binding stability assay

Urea-induced denaturation was used to evaluate the stability of peptide-HLA complexes as described before (18). Briefly, peptides were dissolved in DMSO and 1mM β-mercaptoethanol and dispensed in 96-well plates at a final concentration of 2 µM. The HLA allele of interest was diluted in assay buffer containing β-2 microglobulin and added to the plate in the range of 2–10 nM to ensure excess of peptides during peptide-HLA complex formation. Each plate also included reference peptides with known stable binding to each allele. Following complex formation, samples were transferred to a 384-well plate and stressed with 8 different urea concentrations ranging from 0–7 M. Plates were then developed using ELISA as previously described (19, 20). For each peptide, binding stability to a given allele was calculated relative to the 100% binding stability of the reference peptide. Peptides with a relative binding stability of 50% or higher were annotated as stable binders.

### 2.9 Peptide/HLA binding affinity assay

The affinity of peptide/HLA binding was determined as described before (21, 22). Briefly, biotinylated HLA Class I heavy chains were denatured and diluted into refolding buffer containing β-mercaptoethanol and varying concentrations of the test/control peptides. The mixtures were incubated with agitation at room temperature for 20–24 hours to enable peptide/HLA complex formation. The solutions containing the complexes were transferred to streptavidin plates and incubated for 30 minutes. Plates were then developed using ELISA. Absorbance measured at 450 nm was graphed against the varying concentrations of the test peptide used. The affinity of peptide/HLA binding was determined by calculating the EC50, defined as the peptide concentration resulting in half saturation. Peptides with an EC50 under 100 nM were classified as high affinity binders.

### 2.10 Data analysis and quantification

For FluoroSpot assays, results were expressed as Spot Forming Units (SFU), calculated as the spot count per million PBMCs. The Stimulation Index (SI) was calculated as the average SFU in test wells/average SFU in DMSO control wells. For assays with fresh PBMCs, an SI of 2 or higher with a minimum of 50 SFU was counted as a positive IFN-γ/TNF-α response. For assays with expanded PBMCs, an SI of 1.4 or higher was set as the cutoff for a positive IFN-γ response. Predicted HLA binding alleles for each peptide were determined using MHCMotifDecon 1.0 as previously described (13, 14, 23). Predicted binding affinities for identified epitopes were determined using NetMHC 4.0 (24). Data tables and heatmaps were created using Excel. Data analysis and graphical representations were performed on Graph Pad Prism 10.

## 3 Results

### 3.1 Direct *ex vivo* FluoroSpot failed to detect AAV9 capsid-specific T cells in seropositive donors

**Figure 1:**
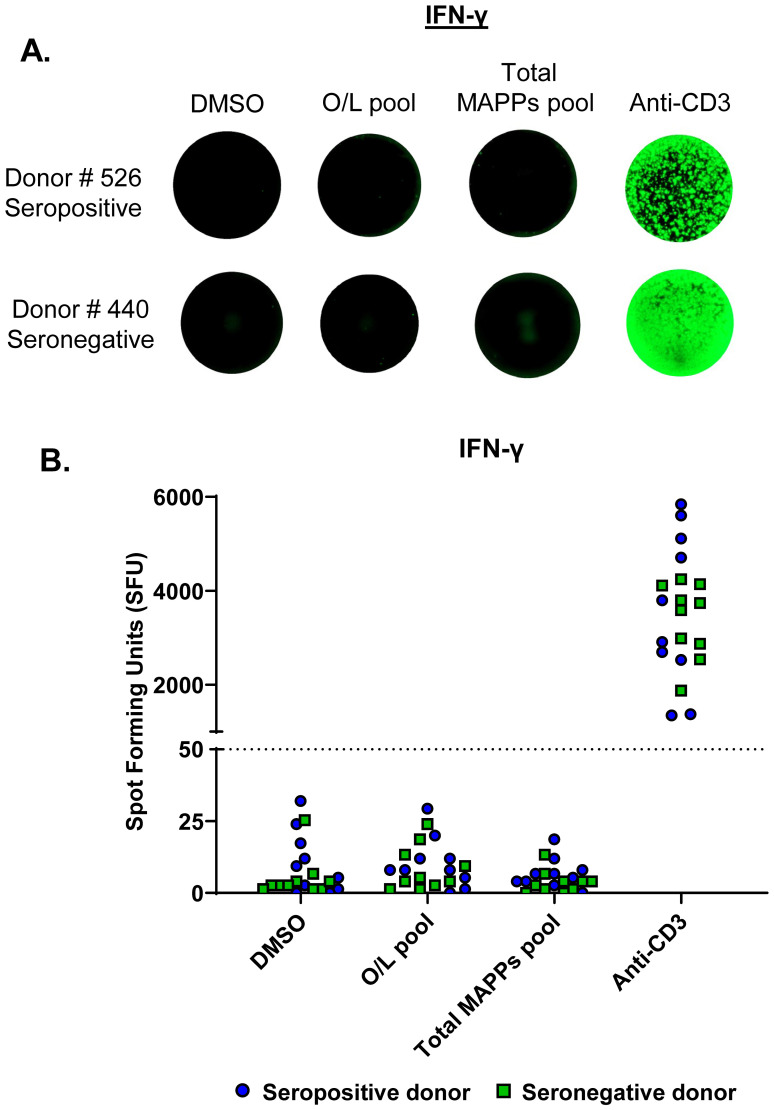
Direct *ex vivo* FluoroSpot failed to detect AAV9 capsid-specific T cells in seropositive donors. PBMCs from AAV9 seropositive (n=10) and seronegative (n=10) healthy donors were stimulated on a FluoroSpot assay to measure IFN-γ secretion in response to peptides derived from the AAV9 capsid protein. **(A)** IFN-γ secretion images for representative donors are shown (one well per condition). Each green spot represents a cytokine-secreting cell. **(B)** IFN-γ secretion data from all tested donors. Spot Forming Units (SFU)- Spot count per million PBMCs; Blue circles- Seropositive donors; Green squares- Seronegative donors; Dotted line- cutoff for positive response (50 SFU). Stimulation pools: Overlapping (O/L) pool (pool of overlapping capsid peptides), total MAPPs pool (pool of all MAPPs-derived capsid peptides). DMSO- negative control; Anti-CD3- positive control antibody; MAPPs, MHC-associated peptide proteomics.

A previous study reported the detection of AAV capsid-specific T cells in PBMCs isolated from AAV2 seropositive healthy donors (25). To determine if we could detect AAV9 capsid-specific T cells in seropositive healthy donors, we first analyzed plasma samples from a cohort of 76 healthy donors for anti-AAV9 antibodies using a total antibody assay (15). We identified 19 seropositive and 57 seronegative donors, of which we selected 10 seropositive and 10 seronegative donors for further analysis (**Supplementary Table 1**). Frozen PBMCs from the 20 selected donors were stimulated for 24 hours on a FluoroSpot assay with either an overlapping peptide pool derived from the AAV9 capsid (15-mers with 11 amino acid overlap, 182 peptides) or a total MAPPs peptide pool containing both HLA Class I and Class II MAPPs-derived peptides of the AAV9 capsid (53 peptides). Circulating AAV9 capsid-specific T cells were detected by measuring IFN-γ and TNF-α release in response to peptide stimulation. We noted a low overall T cell frequency in all tested donors, with no significant detection of capsid-specific T cells in response to either peptide pool (**Figures 1A, B**). Importantly, there was no differentiation in the cytokine response to AAV9 capsid peptides between seropositive and seronegative donors (**Figure 1B**, **Supplementary Figure 1**). This lack of connectivity between anti-AAV antibody and T cell response is best exemplified by Donor # 526 which had the highest antibody signal among all analyzed donors yet lacked T cell response to either overlapping or MAPPs-derived peptide pools (**Figure 1A**, **Supplementary Table 1**). Overall, irrespective of donor serostatus, healthy donor PBMCs appeared to have low numbers of circulating capsid-specific T cells, which resulted in poor sensitivity on the FluoroSpot assays.

### 3.2 Overlapping and MAPPs-derived peptides of the AAV9 capsid expand distinct T cell populations

**Figure 2:**
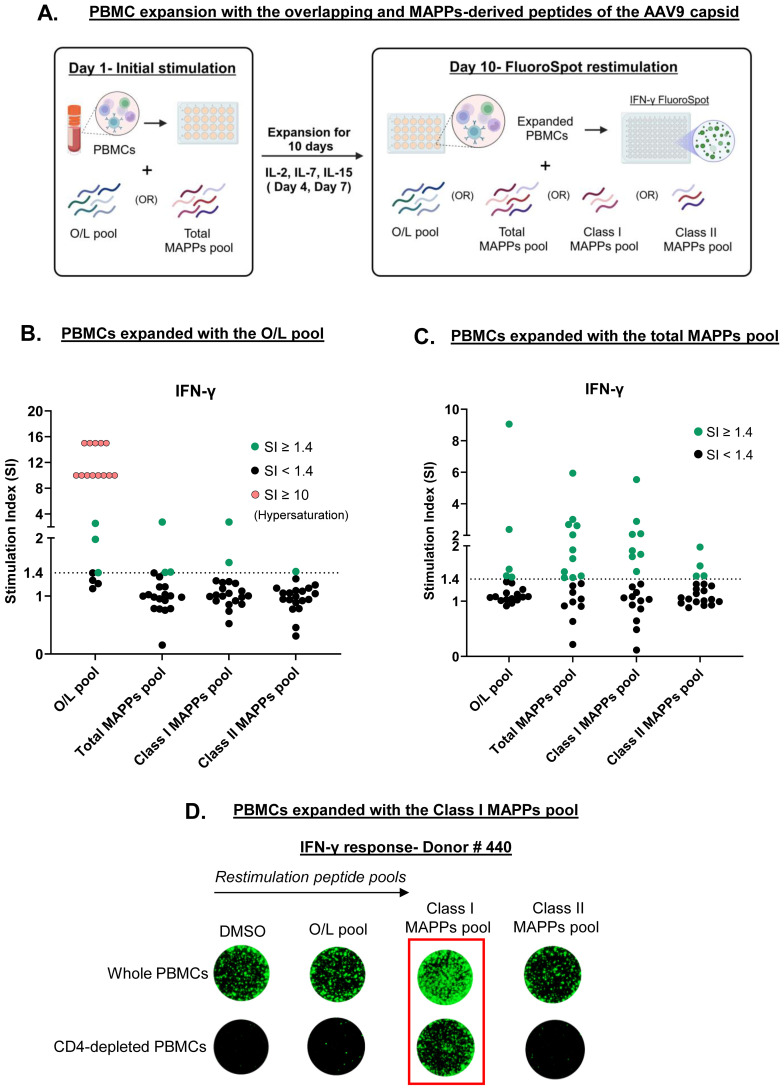
Overlapping and MAPPs-derived peptides of the AAV9 capsid expand distinct T cell populations. **(A)** PBMCs from 20 healthy donors were expanded for 10 days with a pool of either overlapping (O/L) or total MAPPs peptides of the AAV9 capsid. Expanded PBMCs were restimulated on a FluoroSpot assay with four different capsid peptide pools to measure IFN-γ secretion. Created with Biorender.com. **(B)** IFN-γ response in PBMCs expanded with the O/L pool. **(C)** IFN-γ response in PBMCs expanded with the total MAPPs pool. Stimulation index (SI)- Average spot forming units in test wells/average spot forming units in control wells; Dotted line- cutoff for positive response (SI of 1.4); Green circles- donors with positive response; black circles- donors with negative response; pink circles- donors with strong positive response denoted by signal hypersaturation (SI artificially scaled to ≥ 10). **(D)** PBMCs from one healthy donor with and without CD4 depletion were expanded for 10 days with the Class I MAPPs pool. Expanded PBMCs were restimulated on a FluoroSpot assay to measure IFN-γ secretion. Representative images are shown (one well per condition). Peptide pools: O/L pool (pool of overlapping capsid peptides), Total MAPPs pool (pool of all MAPPs-derived capsid peptides), Class I MAPPs pool (pool of MAPPs-derived capsid peptides presented on HLA Class I), Class II MAPPs pool (pool of MAPPs-derived capsid peptides presented on HLA Class II). DMSO-negative control; MAPPs- MHC-associated peptide proteomics.

To increase the frequency of capsid-specific T cells in healthy donors, we adopted a PBMC expansion protocol, wherein PBMCs from 20 healthy donors were expanded with the overlapping or the total MAPPs capsid peptide pools and a cocktail of cytokines for 10 days prior to restimulation on FluoroSpot assays. Expanded PBMCs were then restimulated with either the overlapping peptide pool, the total MAPPs peptide pool, the Class I MAPPs peptide pool (41 HLA Class I MAPPs capsid peptides), or the Class II MAPPs pool (12 HLA Class II MAPPs capsid peptides) (**Figure 2A**). Through PBMC expansion, we sufficiently improved the detection of capsid-specific T cells in healthy donors and were also able to compare the T cell populations expanded by the overlapping and MAPPs-derived capsid peptides.

PBMCs expanded with the overlapping peptide pool displayed a strong IFN-γ response when restimulated with the same pool of peptides. A positive response was seen in 80% of tested donors, with a number of donors showing a hypersaturated response on the FluoroSpot assay indicating strong T cell activation (**Figure 2B**, first column, 16 in 20 positive responders). Conversely, little to no IFN-γ response was observed in these PBMCs upon restimulation with any of the MAPPs-derived peptide pools (**Figure 2B**, remaining columns). In PBMCs expanded with the total MAPPs peptide pool, only 25% of tested donors displayed a positive IFN-γ response upon restimulation with the overlapping peptide pool (**Figure 2C**, first column, 5 in 20 positive responders). In contrast, 55% of tested donors displayed a positive IFN-γ response when restimulated with the total MAPPs pool (**Figure 2C**, second column, 11 in 20 positive responders). Interestingly, the restimulation response to the total MAPPs pool did not correlate with the serostatus of the tested donors (**Supplementary Figure 2**). Furthermore, we observed a distinction in the restimulation response to HLA Class I vs. Class II MAPPs peptides. 40% of tested donors displayed a positive IFN-γ response to the Class I MAPPs pool (**Figure 2C**, third column, 8 in 20 positive responders) compared to 20% of tested donors that responded to the Class II MAPPs pool (**Figure 2C**, fourth column, 4 in 20 positive responders). Overall, our results suggest that overlapping and MAPPs-derived capsid peptides expand distinct T cell populations in healthy donors and that the response to the total MAPPs pool is dominated by the Class I MAPPs pool, suggesting a preferential expansion of capsid-specific CD8 T cells.

To confirm the prevalence of CD8 T cells in the response to the HLA Class I MAPPs peptides, CD4 T cells were depleted from PBMCs of a responding donor prior to expansion with the Class I MAPPs pool and restimulation with either MAPPs-derived or overlapping peptides. Consistent with our previous observations, no response was observed to the overlapping or the Class II MAPPs pools, irrespective of the presence of CD4 T cells. In contrast, restimulation with Class I MAPPs pool elicited robust IFN-γ release in PBMCs even in the absence of CD4 T cells (**Figure 2D**). Overall, our findings confirm that the response to HLA Class I MAPPs peptides is dominated by CD8 T cells.

### 3.3 Peptide deconvolution of the HLA Class I MAPPs peptides reveals previously undefined immunodominant CD8 T cell epitopes

To identify the immunodominant CD8 T cell epitopes within the HLA Class I MAPPs peptides, we utilized a system of matrix pools which facilitates rapid mapping of immunodominant epitopes when investigating a large number of peptides. The 41 HLA Class I MAPPs peptides were arranged into 13 matrix pools (MPs), each containing 6–7 peptides (**Supplementary Table 4**). The matrix arrangement allows each Class I peptide to feature in two MPs, and a restimulation response to both MPs containing a specific peptide identifies putative immunodominant peptides that can be verified on an individual basis. Peptide deconvolution was performed on a cohort of 24 healthy donors. PBMCs were depleted of CD4 T cells and expanded as described before with the Class I MAPPs pool (41 peptides). Expanded PBMCs were restimulated on FluoroSpot assays with the 13 MPs. The putative immunodominant peptides identified through responding MPs were individually analyzed in a separate experiment to confirm their ability to generate a CD8 T cell response.

**Figure 3:**
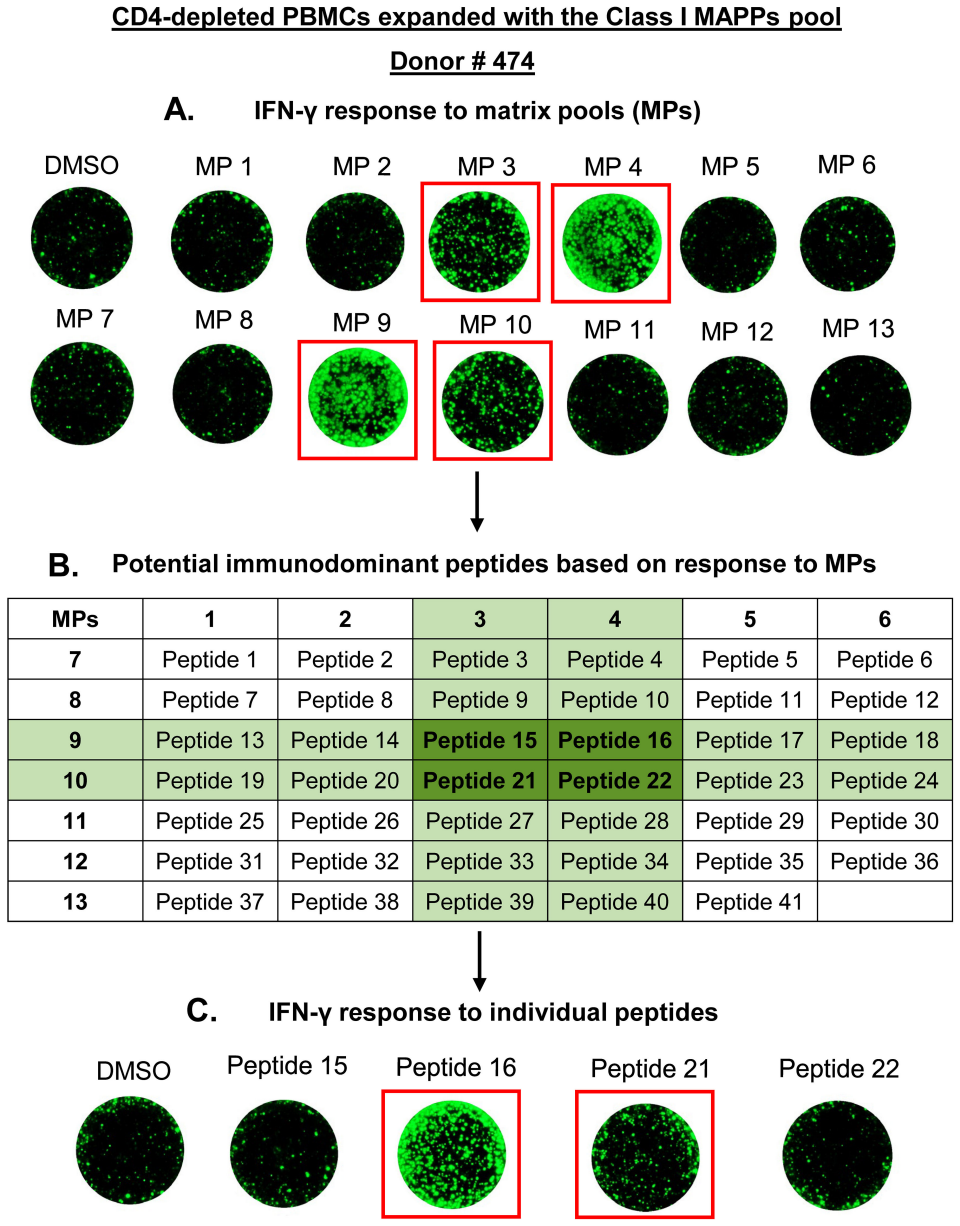
Peptide deconvolution of HLA Class I MAPPs peptides to identify immunodominant CD8 T cell epitopes of the AAV9 capsid. PBMCs from 24 healthy donors were depleted of CD4 T cells and expanded for 10 days with a pool of Class I MAPPs peptides of the AAV9 capsid. Expanded PBMCs were restimulated on a FluoroSpot assay for 24 hours with matrix pools (MPs) as described in **Supplementary Table 4**. Based on positive IFN-γ response to MPs, candidate immunodominant peptides for each donor were shortlisted using the matrix. Each candidate peptide was then tested individually for immunodominance in a subsequent expansion experiment. Representative data from one donor for peptide deconvolution is shown. **(A)** IFN-γ secretion after stimulation by MPs (one well per condition). **(B)** Shortlisting candidate immunodominant peptides using the matrix. **(C)** IFN-γ secretion in response to candidate individual peptides (one well per condition). DMSO- negative control; MAPPs, MHC-associated peptide proteomics.

**Figure 3** describes the peptide deconvolution process for one donor. CD4-depleted PBMCs from Donor # 474 were expanded with the Class I MAPPs pool and restimulated on a FluoroSpot assay with 13 MPs. This donor displayed an IFN-γ response to four MPs- MP 3, MP 4, MP 9, and MP 10 (**Figure 3A**). Based on the matrix, we identified the common peptides between the four responding MPs and shortlisted four candidate immunodominant peptides for this donor (**Figure 3B**). In a subsequent experiment, CD4-depleted PBMCs from Donor # 474 were expanded with the Class I MAPPs pool and restimulated on a FluoroSpot assay with the four candidate immunodominant peptides individually. IFN-γ response was observed to Peptide 16 and Peptide 21, thereby verifying their immunodominance in this donor (**Figure 3C**). **Supplementary Tables 5, 6** contain the summary of the deconvolution data for all peptides and donors.

**Figure 4:**
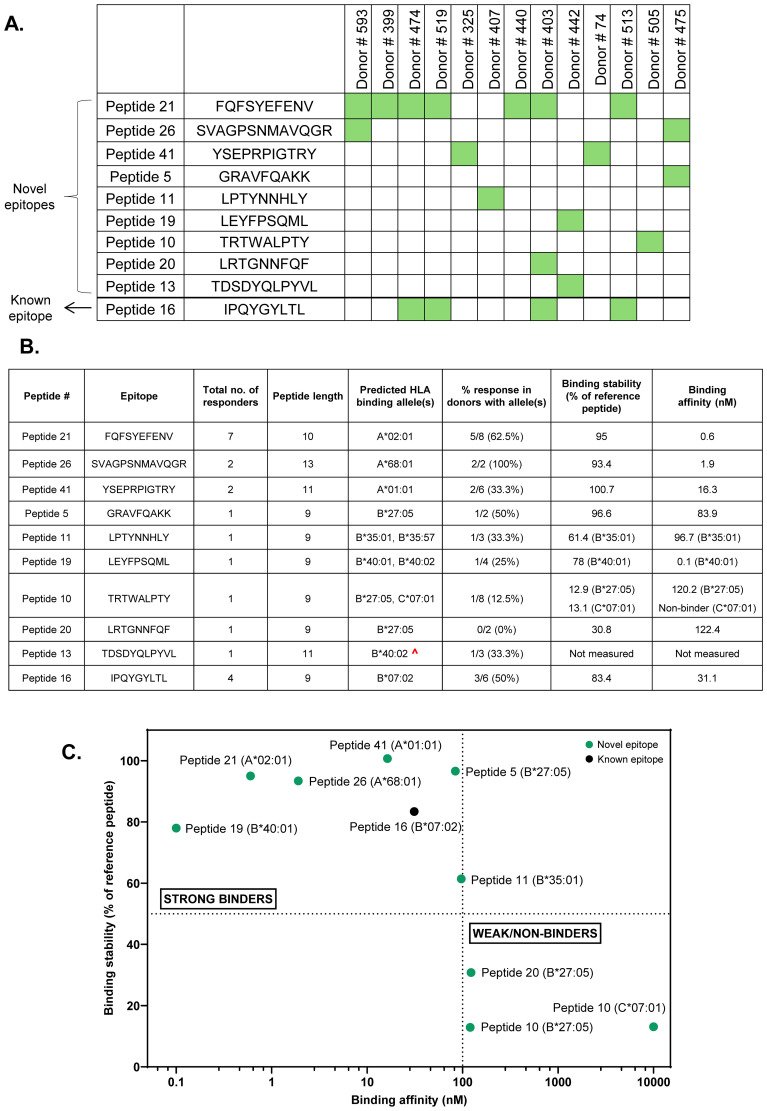
Summary of immunodominant CD8 T cell epitopes identified through peptide deconvolution of HLA Class I MAPPs peptides. **(A)** Map describing Class I MAPPs peptides that elicited a positive IFN-γ response in at least one donor. **(B)** Table describing the length, predicted HLA binding alleles, allele-specific response, and binding parameters for immunodominant epitopes identified in the study. ^- Low confidence allele prediction. **(C)** Classification of identified immunodominant peptides as strong and weak/non-binders based on allele binding stability and affinity. Dotted line on Y axis- minimum cutoff for stable binders (50% of reference peptide); Dotted line on X axis- maximum cutoff for high-affinity binders (100 nM).

Our analysis revealed 10 Class I MAPPs peptides that displayed an IFN-γ response in at least one donor. These included 1 previously reported and 9 novel capsid T cell epitopes (**Figure 4A**, **Supplementary Table 5**). Among the novel epitopes, responses were most prevalent to Peptide 21 (7 responders), followed by Peptide 26 and Peptide 41 (2 responders each), with the remaining 6 peptides having one responding donor each (**Figure 4B**, third column). The novel peptides display significant diversity in their lengths, varying between 9 and 13 amino acids (**Figure 4B**, fourth column). Furthermore, two of the novel peptides- Peptide 21 and Peptide 26- displayed a high percentage of responses in donors expressing their predicted HLA Class I binding alleles (13, 23). Peptide 21 elicited a response in 62.5% of donors with its predicted binding allele (5 responders among 8 donors with the A*02:01 allele) (**Figure 4B**, columns 5, 6). It also elicited responses in two donors with the closely related A*02:02 and A*02:05 alleles (Donor # 399, Donor # 440, **Supplementary Table 6**). Similarly, Peptide 26 elicited a response in 100% of donors with its predicted binding allele (2 responders among 2 donors with the A*68:01 allele). The remaining novel peptides, on the other hand, did not display similar trends and had lower response rates in donors with their predicted binding alleles (**Figure 4B**, columns 5, 6). We also observed T cell responses to the known epitope Peptide 16 in 4 donors. Given its reported immunodominance, this peptide was individually tested in all donors with its predicted binding allele irrespective of whether or not a response was observed to the corresponding matrix pools. Peptide 16 elicited a response in only 50% of the donors with its predicted binding allele (3 responders among 6 with the B*07:02 allele) (**Figure 4B**, row 10).

To further validate the identified CD8 T cell epitopes, we measured the binding stability and affinity of the epitopes to their predicted HLA binding alleles (**Figure 4B**, columns 7, 8, **Figure 4C**). The majority of the identified AAV9 capsid CD8 T cell epitopes were strong binders (high stability and affinity to their respective alleles). Two novel epitopes- Peptide 10 and Peptide 20- have weak binding to their predicted alleles, suggesting that their presenting alleles were not correctly predicted. The measured binding affinities of most epitopes also correlated strongly with the predicted affinities generated using NetMHC (**Supplementary Table 7**). The novel 13-mer epitope Peptide 26 elicited a weak NetMHC rank score despite a high measured binding affinity, which is unsurprising given that the NetMHC model is curated for peptides ranging between 8–11 amino acids (24).

### 3.4 Lack of responses to previously reported immunodominant AAV9 capsid epitopes

In addition to Peptide 16, the HLA Class I MAPPs peptides included 3 other peptides that have been previously reported as immunodominant AAV9 capsid epitopes - Peptide 24, Peptide 7, and Peptide 17 (26, 27). Surprisingly, these peptides did not elicit a response in any tested donors, including the donors with the predicted HLA binding allele (**Table 1**). Notably, Peptide 24 demonstrated strong binding stability and affinity to its predicted A*02:01 allele (**Table 1**, columns 7, 8) but remained a non-responder despite individual testing in seven different donors with the allele (Donors # 403, # 406, # 474, # 505, # 513, # 519, # 593, **Supplementary Table 6**). This presents a sharp contrast to the novel epitope Peptide 21, which also binds strongly to the A*02:01 allele and elicits responses in multiple donors with the allele as previously described.

**Table 1:** Previoulsy described capsid epitopes that did not elicit a T cell response in the current study.

Peptide #	Epitope	Total no. of responders	Peptide length	Predicted HLA binding allele(s)	% response in donors with allele(s)	Binding stability (% of reference peptide)	Binding affinity (nM)
Peptide 24	LIDQYLYYL	0	9	A*02:01	0/8 (0%)	70.2	0.6
Peptide 7	QPAKKRLNF	0	9	B*07:02	0/6 (0%)	Not measured	Not measured
Peptide 17	SQAVGRSSF	0	9	B*15:01	0/2 (0%)	Not measured	Not measured

Overall, despite comparable binding parameters, many of the identified novel epitopes display stronger abilities to induce the capsid-specific CD8 T cell response compared to previously described epitopes.

### 3.5 CD8 T cells preferentially respond to longer T cell epitopes within the same peptide cluster

**Figure 5:**
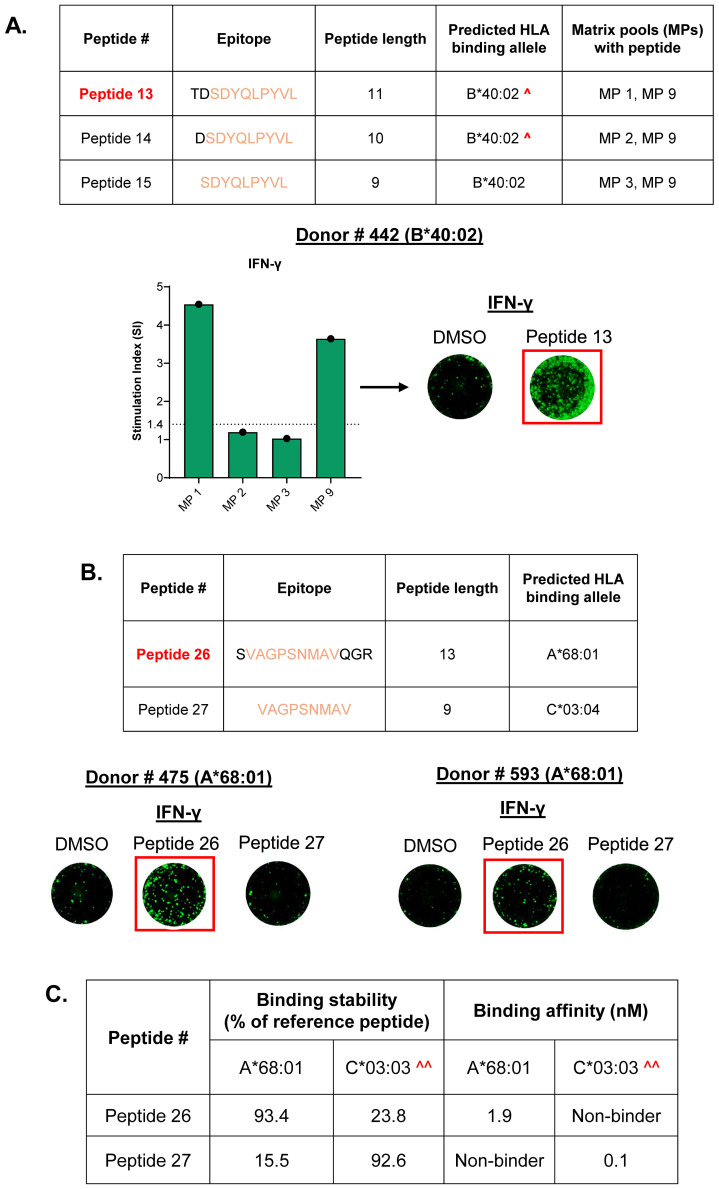
CD8 T cells preferentially respond to longer T cell epitopes within the same peptide cluster. **(A)** Peptide cluster comprising 9-mer, 10-mer, and 11-mer peptides that are predicted to bind to the same HLA allele, along with matrix pools (MPs) with each peptide. IFN-γ response is observed only to the 11-mer Peptide 13, denoted by responses to both the corresponding matrix pools and the individual peptide. **^**- Low confidence allele prediction. **(B)** Peptide cluster comprising 9-mer and 13-mer peptides along with the predicted HLA binding alleles. Donors with the A*68:01 allele display IFN-γ response only to the 13-mer Peptide 26 when tested individually. Stimulation index (SI)- Average spot forming units in test wells/average spot forming units in control wells; Dotted line- cutoff for positive response (SI of 1.4); Representative FluoroSpot images are shown (one well per condition); DMSO-negative control. **(C)** Binding stabilities and affinities of clustered Peptide 26 and Peptide 27 to their predicted binding alleles. Minimum cutoff for stable binders- 50% of reference peptide; Maximum cutoff for high-affinity binders- 100nM; **^^**- Binding was measured to the closely related C*03:03 allele as a substitute for the C*03:04 allele.

Many of the identified HLA Class I MAPPs peptides were organized as peptide clusters, each comprising 2–3 peptides of varying lengths and the same peptide core (**Supplementary Table 8**). Of these, T cell responses were observed to peptides belonging to 2 clusters. The first cluster contained 9-mer (Peptide 15), 10-mer (Peptide 14), and 11-mer (Peptide 13) peptides that were presented by the same donor on the MAPPs assay (**Supplementary Table 8**, Cluster 3). All three peptides were predicted to bind to the B*40:02 allele, but the rank score threshold for accurate prediction was met only for the 9-mer Peptide 15 (13). Interestingly, despite having a non-significant prediction score, only the 11-mer Peptide 13 elicited a T cell response in a donor expressing the B*40:02 allele (**Figure 5A**). The second cluster contained 9-mer (Peptide 27) and 13-mer (Peptide 26) peptides that were presented by two different donors on the MAPPs assay and had two different predicted binding alleles (**Supplementary Table 8**, Cluster 4). Here, the 13-mer Peptide 26 elicited a T cell response in donors containing its predicted binding allele (A*68:01). Peptide 27, on the other hand, did not elicit a response in any donors with its predicted binding allele C*03:04 (**Figure 5B**, **Supplementary Figure 3**). Binding assays indicate stable and high affinity binding of both peptides to their respective predicted or closely related alleles (**Figure 5C**). This indicates that, even with comparable binding stabilities, longer epitopes are more likely to induce T cell activation.

Taken together, our peptide deconvolution experiments identified several immunodominant CD8 T cell epitopes, of which only one was previously reported. A majority of the identified epitopes are novel peptides of varying lengths that display high response rates in donors with the predicted binding alleles. Binding assays further confirmed stable binding and strong affinity of novel epitopes to their predicted binding alleles. Finally, we observed that longer epitopes within the same peptide cluster are preferred for CD8 T cell activation.

## 4 Discussion

One of the major drawbacks of AAV-based gene therapies is the cellular immune response to the capsid protein of AAV. Specifically, the capsid-specific CD8 T cells are capable of eliminating transduced cells, leading to the loss of transgene expression (7–9, 28). In the current study, we analyzed the T cell responses to the naturally presented capsid peptides of the AAV9 capsid protein. Identified through MAPPs, these peptides represent a functionally relevant system to monitor the capsid-specific T cell response in patients. Additionally, through the analysis of peptides displayed uniquely on HLA Class I, we identified immunodominant capsid epitopes that contribute to the CD8 cytotoxic T cell response.

Our initial attempts to measure the AAV9 capsid-specific T cell response focused on using fresh PBMCs isolated from healthy donors. A similar study in AAV2 noted an important role for preexisting AAV immunity, where researchers observed distinct cytokine secretion in T cells from donors with and without preexisting anti-AAV antibodies (25). In our assays, however, T cell responses were largely undetectable in healthy donors regardless of preexisting immunity. The issue of low baseline T cell frequency has been a common observation in studies that evaluate viral antigens using healthy donors (29, 30). This has typically been addressed by performing an *in vitro* expansion of PBMCs to enable the proliferation of antigen specific T cells (29–31). In the context of AAV, this approach has been successfully employed to improve the detection of capsid-specific T cells and formed as the basis for our subsequent analyses (32, 33).

The current approach to monitoring the capsid-specific T cell response involves the use of overlapping peptides spanning the entire capsid protein. However, these assays have low sensitivity and are often unable to detect capsid-specific T cells even in individuals with a high titer of anti-capsid antibodies (34, 35). In our study, we observed that overlapping and MAPPs-derived peptides are recognized by and stimulate the activation of largely distinct T cell populations in healthy donors. T cells expanded with the naturally presented MAPPs-derived peptides were not efficiently restimulated by overlapping peptides and, conversely, T cells expanded with overlapping peptides did not respond to MAPPs-derived peptides. The precise target(s) of the T cell responses elicited by the overlapping peptides in healthy donors in our assay is not clear but a recent study from Bing et al., 2023, uncovered, using overlapping peptides, an immunodominant HLA-DP-restricted CD4 T cell epitope in the AAV9 capsid (36). This T cell epitope was absent from our MAPPs-derived peptide pools since the HLA Class II immunopeptidome of AAV9 was predominantly restricted to HLA-DR and HLA-DQ, with no significant presentation on HLA-DP (14). In contrast, the T cell responses to the MAPPs-derived peptides was dominated by CD8 T cells recognizing peptides eluted from HLA Class I, perhaps reflecting the fact that, unlike Class I MAPPs peptides, 15-mer overlapping peptides may require additional processing before they can bind to HLA Class I molecules and elicit CD8 T cell responses.

Several techniques have been previously employed to identify and characterize capsid epitopes that activate CD8 T cells, including overlapping capsid peptides (26, 37), MHC multimers (8, 27, 38), and *in silico* binding predictions (39). For the first time, we leveraged capsid peptides derived from HLA Class I to identify 9 novel immunodominant CD8 epitopes of the AAV9 capsid. The discovery of novel epitopes highlights the importance of evaluating naturally presented capsid peptides, as these peptides remain unidentified through previously employed means of epitope identification. Binding assays confirmed that 6 of the 9 epitopes displayed strong binding to their predicted alleles. Among the identified novel epitopes, Peptide 21 (FQFSYEFENV) elicited a response in the highest number of donors and exhibits strong binding to the HLA-A*02:01 allele, which is one of the most common alleles that is present in ~20% of the US population. The knowledge of the novel epitopes, thus, has the potential to impact a broad range of the population. Interestingly, the Class I MAPPs pool included another HLA-A*02:01-restricted epitope Peptide 24 (LIDQYLYYL), that was previously identified as an immunodominant epitope from AAV capsids (26, 27). Our binding assays confirmed that Peptide 24 binds to the A*02:01 allele with an affinity similar to that of Peptide 21 (0.6 nM), but unlike Peptide 21, Peptide 24 did not elicit responses in any of the donors with the A*02:01 allele that were tested in this study. The varied response in A*02:01 donors to two equally strong binders could be driven by a difference in precursor frequency in healthy individuals, which has been reported to be a distinguishing factor between immunodominant and subdominant epitopes in the context of SARS-CoV-2 (40). This would explain the superior T cell response to Peptide 21 in our study after just a single round of *in vitro* expansion. In contrast, a previous study reported performing up to three rounds of restimulation to detect a response to Peptide 24 (26).

Epitope mapping relying on overlapping peptides or *in silico* binding prediction tools are often focused on identifying 9-mer peptides, the most common length found in natural HLA Class I ligands. The naturally processed CD8 T cell epitopes identified in the current study exhibit more diverse lengths, ranging from 9 to 13 amino acids. We have confirmed that the longest epitope identified in the current study, the 13-mer Peptide 26 (SVAGPSNMAVQGR) displayed strong binding to HLA-A*68:01, explaining the high response rate in donors expressing this allele. The shorter overlapping 9-mer Peptide 27 (VAGPSNMAV), on the other hand, binds to a different allele but did not elicit a response in the corresponding donors. This preferential binding of atypical long CD8 T cell peptides over an overlapping shorter sequences has been reported for HLA-A alleles- HLA-A*02:01 (41), as well as HLA-B alleles- HLA-B*35 (42, 43), HLA-B*07:02 (44), HLA-B*44:03 (45), suggesting that the peptide length specificity of some HLA Class I alleles is broad, allowing for longer peptides to dominate over Class I ligands of canonical length as targets for T cell recognition. The structural basis for the high immunogenicity of longer peptides is still poorly understood but could be explained by the inherent plasticity of T cell receptors (TCRs) that allows flexibility at the TCR-pHLA-I interface to enable the recognition of longer epitopes protruding out of the HLA binding groove (44).

At present, methods used to monitor cellular immunity to AAV-based gene therapies in clinic rely on the use of overlapping peptides derived from AAV capsids or transgene. We believe that the incorporation of naturally processed T cell epitopes of AAV capsids such as the one we uncovered in this study will enhance our capacity to reliably detect capsid-specific T cells in patients. A similar push to characterize naturally processed T cell epitopes from the Cas9 editing enzyme is currently underway in the field of gene editing (12, 46). Ultimately, these naturally processed immunodominant peptides offer new targets for vector engineering, where the mutation or elimination of these sequences may aid in circumventing the CD8 cytotoxic T cell response (36, 47, 48).

## Data Availability

The original contributions presented in the study are included in the article/**Supplementary Material**. further inquiries can be directed to the corresponding authors.
