# Supplementary material for: Epitope mapping using immunopeptidomics reveals novel immunodominant CD8 T cell epitopes of the AAV9 capsid
Source: Front Immunol. 2025 Aug 8;16:1641289. doi: 10.3389/fimmu.2025.1641289 (PMC12371361; doi:10.3389/fimmu.2025.1641289)
Supplement: Supplementary file 1 [file DataSheet1.pdf]

**Epitope mapping using immunopeptidomics reveals novel immunodominant CD8 T cell epitopes of the AAV9 capsid**

Akhila Balasubramanian[1], Marek Prachar[2], Birgit Klaproth[2], Victoria Copeland[1], Sune Justesen[2], Yi Wen[1], Robert W. Siegel[1*], Laurent P. Malherbe[1*]

[1]Lilly Research Laboratories, Eli Lilly and Company, Indianapolis, IN., United States

[2]Lilly Oncology, Eli Lilly and Company, Copenhagen, Denmark

[*]Corresponding Author

Correspondence: siegel_robert@lilly.com , malherbe_laurent@lilly.com

**SUPPLEMENTAL INFORMATION**

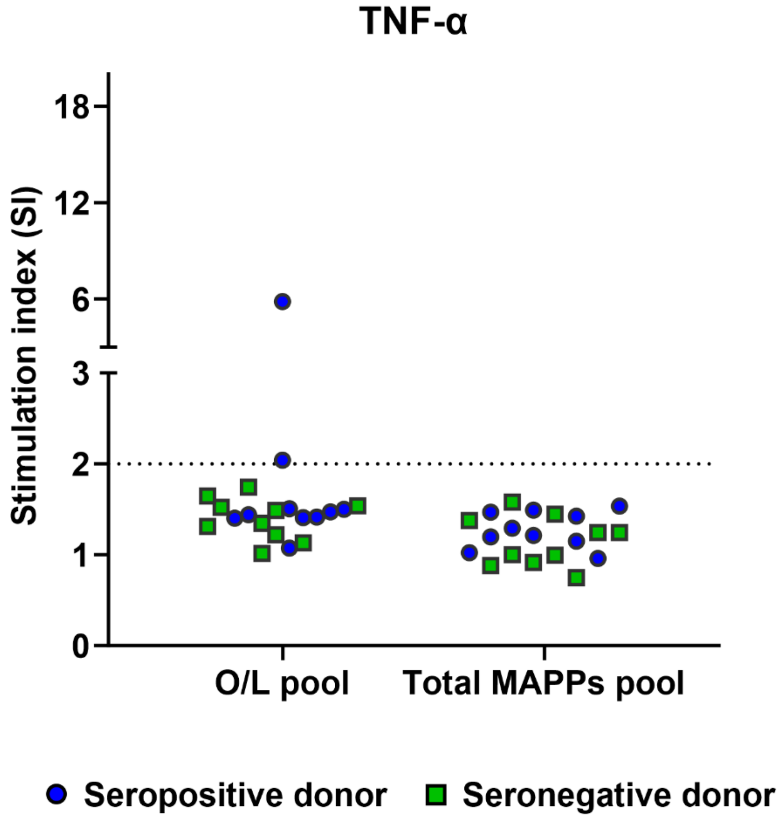


**Supplemental Figure 1: TNF-α secretion in AAV9 capsid-specific T cells is undetectable in seropositive donors.** PBMCs from AAV9 seropositive (n=10) and seronegative (n=10) healthy donors were stimulated on a FluoroSpot assay to measure TNF-α secretion in response to peptides derived from the AAV9 capsid protein. Blue circles- Seropositive donors; Green squares- Seronegative donors; Stimulation index (SI)- Average spot forming units in test wells/ average spot forming units in control wells; Dotted line- cutoff for positive response (SI of 2). Stimulation pools: Overlapping (O/L) pool (pool of overlapping capsid peptides), total MAPPs pool (pool of all MAPPs-derived capsid peptides). MAPPs- MHC-associated peptide proteomics.

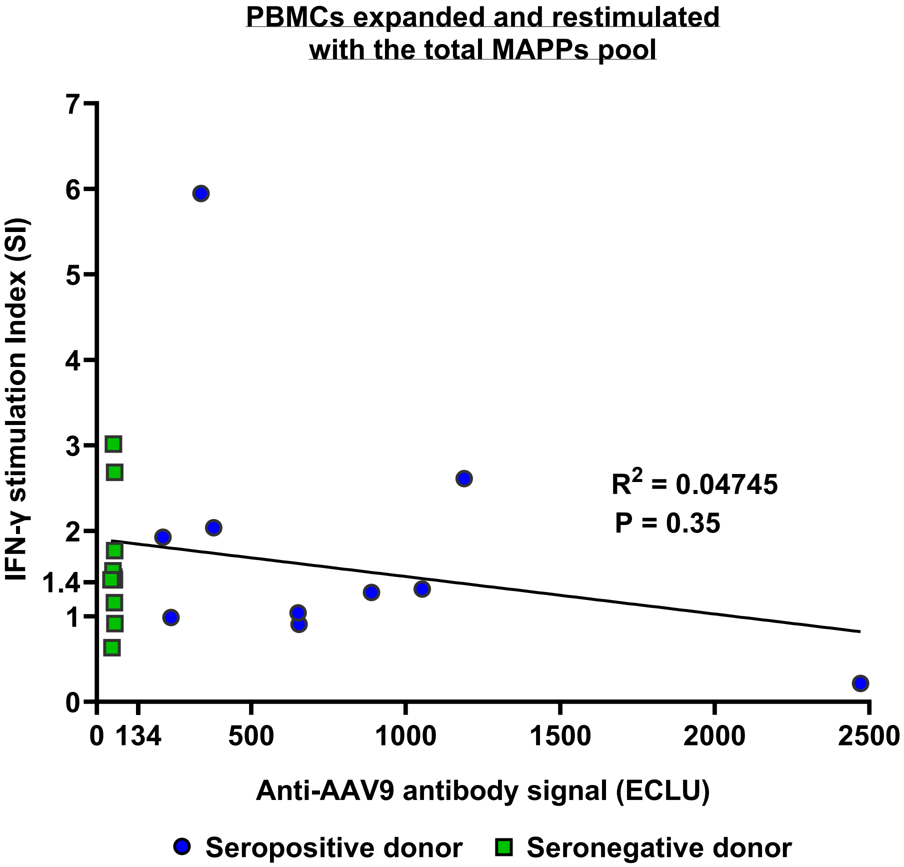


**Supplemental Figure 2: T cell response to MAPPs peptides does not correlate with donor serostatus.** PBMCs from 20 healthy donors (10 AAV9 seropositive and 10 AAV9 seronegative donors) were expanded and restimulated with a pool of the total MAPPs peptides of the AAV9 capsid to measure IFN-γ secretion. Blue circles- Seropositive donors; Green squares- Seronegative donors; Stimulation index (SI)- Average spot forming units in test wells/ average spot forming units in control wells; Cutoff for positive IFN-γ response- SI of 1.4; Anti-AAV9 total antibody assay Tier 1 cut point- 134 ECLU; $R^2$ and P values were determined using Pearson correlation. MAPPs- MHC-associated peptide proteomics.

**A.**

| Peptide # | Epitope | Peptide length | Predicted HLA binding allele | Matrix pools (MPs) with peptide |
|---|---|---|---|---|
| Peptide 26 | SVAGPSNMAVQGR | 13 | A*68:01 | MP 2, MP 11 |
| Peptide 27 | VAGPSNMAV | 9 | C*03:04 | MP 3, MP 11 |

**B.**

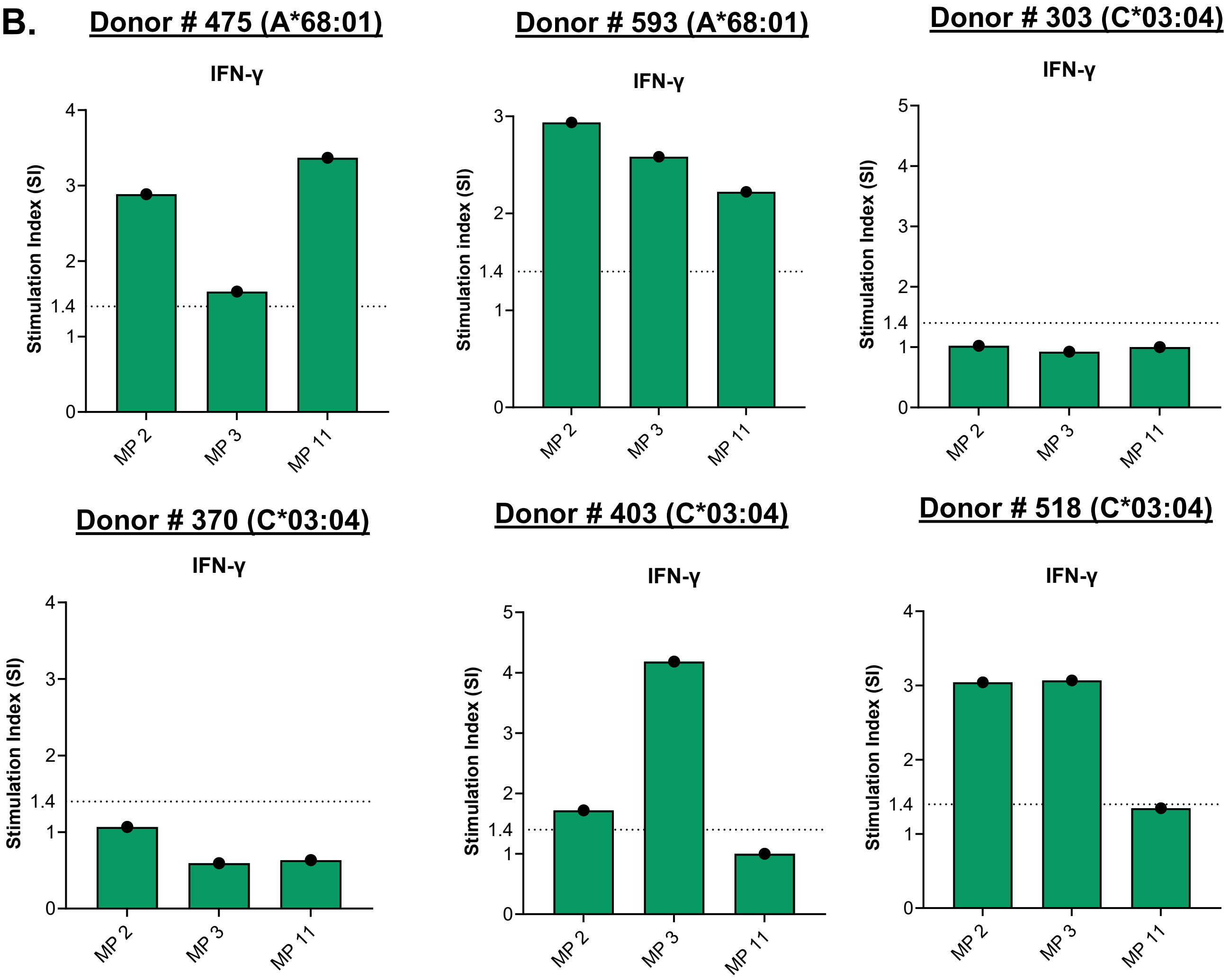


**Supplemental Figure 3: Clustered peptides demonstrate differential T cell response in donors with the predicted binding alleles. A.** Peptide cluster comprising 9-mer and 13-mer peptides, along with matrix pools with each peptide and the predicted HLA binding alleles. **B.** IFN-γ response to matrix pools in donors containing the predicted HLA binding alleles for Peptide 26 (A*68:01) and Peptide 27 (C*03:04). Only matrix pools containing Peptide 26 elicit a response in donors with the appropriate binding allele. Stimulation index (SI)- Average spot forming units in test wells/ average spot forming units in control wells; Dotted line- cutoff for positive response (SI of 1.4).

**Supplemental Table 1: Measurement of anti-AAV9 antibodies in healthy donor plasma**

| Donor # | Anti-AAV9 antibody signal (ECLU) | Serostatus |
|---|---|---|
| 202 | 46 | Seronegative |
| 455 | 46.5 | Seronegative |
| 519 | 49 | Seronegative |
| 369 | 52.5 | Seronegative |
| 398 | 52.5 | Seronegative |
| 408 | 54 | Seronegative |
| 302 | 54.5 | Seronegative |
| 405 | 55.5 | Seronegative |
| 406 | 55.5 | Seronegative |
| 413 | 55.5 | Seronegative |
| 414 | 55.5 | Seronegative |
| 421 | 55.5 | Seronegative |
| 364 | 56 | Seronegative |
| 415 | 56 | Seronegative |
| 262 | 57 | Seronegative |
| 418 | 57 | Seronegative |
| 514 | 57 | Seronegative |
| 543 | 57 | Seronegative |
| 440 | 57.5 | Seronegative |
| 303 | 58 | Seronegative |
| 553 | 58 | Seronegative |
| 280 | 58.5 | Seronegative |
| 515 | 58.5 | Seronegative |
| 434 | 59 | Seronegative |
| 557 | 59.5 | Seronegative |
| 251 | 60 | Seronegative |
| 529 | 60 | Seronegative |
| 530 | 60.5 | Seronegative |
| 539 | 60.5 | Seronegative |
| 383 | 61 | Seronegative |
| 541 | 61 | Seronegative |
| 354 | 61.5 | Seronegative |
| 313 | 61.5 | Seronegative |
| 549 | 62 | Seronegative |
| 552 | 62 | Seronegative |

| | | |
|---|---|---|
| 419 | 62.5 | Seronegative |
| 458 | 62.5 | Seronegative |
| 385 | 63 | Seronegative |
| 547 | 63 | Seronegative |
| 475 | 63.5 | Seronegative |
| 548 | 65.5 | Seronegative |
| 402 | 67.5 | Seronegative |
| 521 | 67.5 | Seronegative |
| 420 | 68.5 | Seronegative |
| 409 | 69.5 | Seronegative |
| 401 | 71 | Seronegative |
| 551 | 71 | Seronegative |
| 436 | 71.5 | Seronegative |
| 399 | 73.5 | Seronegative |
| 416 | 76 | Seronegative |
| 431 | 79 | Seronegative |
| 443 | 105.5 | Seronegative |
| 520 | 109 | Seronegative |
| 376 | 111 | Seronegative |
| 550 | 111 | Seronegative |
| 387 | 113.5 | Seronegative |
| 456 | 133.5 | Seronegative |
| 382 | 134.5 | Seronegative |
| 407 | 214.5 | Seropositive |
| 371 | 240 | Seropositive |
| 351 | 274 | Seropositive |
| 410 | 296 | Seropositive |
| 268 | 336 | Seropositive |
| 513 | 337.5 | Seropositive |
| 403 | 378.5 | Seropositive |
| 546 | 382 | Seropositive |
| 356 | 433 | Seropositive |
| 362 | 467 | Seropositive |
| 227 | 612 | Seropositive |
| 370 | 652 | Seropositive |
| 74 | 655 | Seropositive |
| 271 | 802 | Seropositive |
| 325 | 889.5 | Seropositive |

| | | |
|---|---|---|
| 474 | 1053.5 | Seropositive |
| 345 | 1189 | Seropositive |
| 526 | 2472 | Seropositive |

Donors selected for initial cohort are highlighted in green (for seronegative) and blue (for seropositive) respectively. The total antibody assay Tier 1 cut point was 134 ECLU.

**Supplemental Table 2: Complete donor cohort utilized in the study**

| Donor # | AAV9 serostatus | HLA-A alleles | HLA-B alleles | HLA-C alleles | Purpose |
|---|---|---|---|---|---|
| 74 | Seropositive | A*01:01<br>A*30:01 | B*08:01<br>B*42:01 | C*07:01<br>C*17:01G | All studies |
| 202 | Seronegative | A*24:02<br>A*31:01 | B*35:01<br>B*39:05 | C*04:01<br>C*07:02 | All studies |
| 303 | Seronegative | A*23:01<br>A*30:04 | B*08:01<br>B*15:17 | C*03:04<br>C*07:01 | All studies |
| 325 | Seropositive | A*01:01<br>A*31:01 | B*08:01<br>B*39:11 | C*07:01<br>C*07:02 | All studies |
| 345 | Seropositive | A*11:01<br>A*24:07 | B*15:21<br>B*35:05 | C*04:01<br>C*04:03 | All studies |
| 369 | Seronegative | A*01:01<br>A*24:14 | B*08:01<br>B*40:02 | C*03:04<br>C*07:01 | Fig 1-Fig 2 studies only |
| 370 | Seropositive | A*01:01<br>A*31:01 | B*08:01<br>B*40:08 | C*03:04<br>C*07:01 | All studies |
| 371 | Seropositive | A*23:01<br>A*30:02 | B*18:01<br>B*53:01 | C*05:01<br>C*06:02 | All studies |
| 399 | Seronegative | A*02:05<br>A*24:02 | B*15:01<br>B*49:01 | C*01:02<br>C*07:01 | Fig 3-Fig 5 studies only |
| 403 | Seropositive | A*02:01<br>A*30:01 | B*40:01<br>B*42:01 | C*03:04<br>C*17:01 | All studies |
| 406 | Seronegative | A*02:01<br>A*26:01 | B*14:01<br>B*35:01 | C*04:01<br>C*08:02 | All studies |
| 407 | Seropositive | A*02:02<br>A*03:01 | B*35:01<br>B*44:02 | C*04:01<br>C*05:01 | All studies |
| 408 | Seronegative | A*01:01<br>A*02:01 | B*44:02<br>B*58:01 | C*05:01<br>C*07:18 | All studies |
| 440 | Seronegative | A*02:02<br>A*30:02 | B*07:02<br>B*15:10 | C*04:01<br>C*07:02 | All studies |
| 442 | N/A | A*03:01<br>A*32:01 | B*08:01<br>B*40:02 | C*02:02<br>C*07:01 | Fig 3-Fig 5 studies only |
| 474 | Seropositive | A*02:01<br>A*03:01 | B*07:02<br>B*44:02 | C*05:01<br>C*07:02 | All studies |
| 475 | Seronegative | A*25:01<br>A*68:01 | B*27:05<br>B*44:02 | C*01:02<br>C*07:04 | Fig 3-Fig 5 studies only |
| 505 | N/A | A*02:01<br>A*32:01 | B*27:05<br>B*44:02 | C*01:02<br>C*05:01 | Fig 3-Fig 5 studies only |
| 513 | Seropositive | A*02:01<br>~ | B*07:02<br>B*18:01 | C*07:02<br>C*07:04 | All studies |
| 514 | Seronegative | A*01:01<br>A*29:02 | B*44:03<br>B*57:01 | C*06:02<br>C*16:01 | All studies |
| 515 | Seronegative | A*03:01<br>A*33:03 | B*07:02<br>B*50:01 | C*06:02<br>C*07:02 | All studies |
| 518 | N/A | A*03:01<br>A*24:02 | B*40:01<br>B*40:02 | C*02:02<br>C*03:04 | Fig 3-Fig 5 studies only |
| 519 | Seronegative | A*02:01<br>A*03:01 | B*07:02<br>B*13:02 | C*06:02<br>C*07:02 | All studies |

| | | | | | |
|---|---|---|---|---|---|
| 526 | Seropositive | A*02:05<br>A*11:01 | B*35:01<br>B*49:01 | C*04:01<br>C*07:01 | Fig 1-Fig 2 studies only |
| 553 | Seronegative | A*02:01<br>A*68:03 | B*39:05<br>B*40:01 | C*03:04<br>C*07:02 | Fig 1-Fig 2 studies only |
| 590 | N/A | A*01:01<br>A*03:01 | B*15:01<br>B*40:02 | C*02:02<br>C*03:03 | Fig 3-Fig 5 studies only |
| 593 | N/A | A*02:01<br>A*68:01 | B*07:02<br>B*44:02 | C*07:02<br>C*07:04 | Fig 3-Fig 5 studies only |

**Supplemental Table 3: List of MAPPs-derived AAV9 capsid peptides evaluated in the study**

| HLA type | Peptide # | Epitope | Peptide length | Start | End |
|---|---|---|---|---|---|
| HLA Class I | Peptide 1 | ARGLVLPGY | 9 | 42 | 50 |
| HLA Class I | Peptide 2 | KEDTSFGGNL | 10 | 105 | 114 |
| HLA Class I | Peptide 3 | DTSFGGNLGR | 10 | 107 | 116 |
| HLA Class I | Peptide 4 | TSFGGNLGR | 9 | 108 | 116 |
| HLA Class I | Peptide 5 | GRAVFQAKK | 9 | 115 | 123 |
| HLA Class I | Peptide 6 | KRLLEPLGL | 9 | 123 | 131 |
| HLA Class I | Peptide 7 | QPAKKRLNF | 9 | 165 | 173 |
| HLA Class I | Peptide 8 | RVITTSTRTW | 10 | 238 | 247 |
| HLA Class I | Peptide 9 | ITTSTRTW | 8 | 240 | 247 |
| HLA Class I | Peptide 10 | TRTWALPTY | 9 | 244 | 252 |
| HLA Class I | Peptide 11 | LPTYNNHLY | 9 | 249 | 257 |
| HLA Class I | Peptide 12 | KLFNIQVKEV | 10 | 316 | 325 |
| HLA Class I | Peptide 13 | TDSDYQLPYVL | 11 | 346 | 356 |
| HLA Class I | Peptide 14 | DSDYQLPYVL | 10 | 347 | 356 |
| HLA Class I | Peptide 15 | SDYQLPYVL | 9 | 348 | 356 |
| HLA Class I | Peptide 16 | IPQYGYLTL | 9 | 374 | 382 |
| HLA Class I | Peptide 17 | SQAVGRSSF | 9 | 386 | 394 |
| HLA Class I | Peptide 18 | RSSFYCLEY | 9 | 391 | 399 |
| HLA Class I | Peptide 19 | LEYFPSQML | 9 | 397 | 405 |
| HLA Class I | Peptide 20 | LRTGNNFQF | 9 | 405 | 413 |
| HLA Class I | Peptide 21 | FQFSYEFENV | 10 | 411 | 420 |
| HLA Class I | Peptide 22 | SSYAHSQSL | 9 | 424 | 432 |
| HLA Class I | Peptide 23 | LMNPLIDQY | 9 | 435 | 443 |
| HLA Class I | Peptide 24 | LIDQYLYYL | 9 | 439 | 447 |
| HLA Class I | Peptide 25 | GQNQQTLKF | 9 | 455 | 463 |
| HLA Class I | Peptide 26 | SVAGPSNMAVQGR | 13 | 464 | 476 |
| HLA Class I | Peptide 27 | VAGPSNMAV | 9 | 465 | 473 |
| HLA Class I | Peptide28 | SEFAWPGASSW | 11 | 499 | 509 |
| HLA Class I | Peptide 29 | FAWPGASSW | 9 | 501 | 509 |
| HLA Class I | Peptide 30 | SLMNPGPAM | 9 | 516 | 524 |
| HLA Class I | Peptide 31 | RFFPLSGSLI | 10 | 533 | 542 |
| HLA Class I | Peptide 32 | RFFPLSGSLIF | 11 | 533 | 543 |

| | | | | | |
|---|---|---|---|---|---|
| HLA Class I | Peptide 33 | QSAQAQAQTGW | 11 | 585 | 595 |
| HLA Class I | Peptide 34 | LPGMVWQDRDVY | 12 | 602 | 613 |
| HLA Class I | Peptide 35 | MVWQDRDVY | 9 | 605 | 613 |
| HLA Class I | Peptide 36 | TPVPADPPTAF | 11 | 652 | 662 |
| HLA Class I | Peptide 37 | VPADPPTAF | 9 | 654 | 662 |
| HLA Class I | Peptide 38 | KLNSFITQY | 9 | 666 | 674 |
| HLA Class I | Peptide 39 | KRWNPEIQY | 9 | 693 | 701 |
| HLA Class I | Peptide 40 | YYKSNNVEF | 9 | 705 | 713 |
| HLA Class I | Peptide 41 | YSEPRPIGTRY | 11 | 721 | 731 |
| | | | | | |
| HLA Class II | Peptide 1 | QERLKEDTSF | 10 | 101 | 110 |
| HLA Class II | Peptide 2 | QERLKEDTSFGGNLGRAVF | 19 | 101 | 119 |
| HLA Class II | Peptide 3 | GGNLGRAVFQAKKRL | 15 | 111 | 125 |
| HLA Class II | Peptide 4 | QAKKRLLEPLGL | 12 | 120 | 131 |
| HLA Class II | Peptide 5 | TDSDYQLPY | 9 | 346 | 354 |
| HLA Class II | Peptide 6 | IDQYLYYLSKTING | 14 | 440 | 453 |
| HLA Class II | Peptide 7 | IDQYLYYLSKTINGSG | 16 | 440 | 455 |
| HLA Class II | Peptide 8 | IDQYLYYLSKTINGSGQ | 17 | 440 | 456 |
| HLA Class II | Peptide 9 | IDQYLYYLSKTINGSGQNQ | 19 | 440 | 458 |
| HLA Class II | Peptide 10 | IPGPSYRQQRVSTTVTQNNNSE | 22 | 479 | 500 |
| HLA Class II | Peptide 11 | AVNTEGVYSEPRPIGTRYLTRNL | 23 | 714 | 736 |
| HLA Class II | Peptide 12 | GTRYLTRNL | 9 | 728 | 736 |

**Supplemental Table 4: Arrangement of HLA Class I MAPPs peptides in a matrix for peptide deconvolution**

| MPs | 1 | 2 | 3 | 4 | 5 | 6 |
|---|---|---|---|---|---|---|
| **7** | Peptide 1 | Peptide 2 | Peptide 3 | Peptide 4 | Peptide 5 | Peptide 6 |
| **8** | Peptide 7 | Peptide 8 | Peptide 9 | Peptide 10 | Peptide 11 | Peptide 12 |
| **9** | Peptide 13 | Peptide 14 | Peptide 15 | Peptide 16 | Peptide 17 | Peptide 18 |
| **10** | Peptide 19 | Peptide 20 | Peptide 21 | Peptide 22 | Peptide 23 | Peptide 24 |
| **11** | Peptide 25 | Peptide 26 | Peptide 27 | Peptide 28 | Peptide 29 | Peptide 30 |
| **12** | Peptide 31 | Peptide 32 | Peptide 33 | Peptide 34 | Peptide 35 | Peptide 36 |
| **13** | Peptide 37 | Peptide 38 | Peptide 39 | Peptide 40 | Peptide 41 | |

MP- Matrix pools (MP 1- MP 13), each containing 6-7 peptides listed in each row or column.

**Supplemental Table 5: Summary of immune responses to HLA Class I MAPPs peptides as identified through peptide deconvolution**

| Peptide # | Epitope | Peptide length | Predicted HLA binding allele(s) | Responding donors |
|---|---|---|---|---|
| Peptide 1 | ARGLVLPGY | 9 | B*27:05 | - |
| Peptide 2 | KEDTSFGGNL | 10 | B*40:01 | - |
| Peptide 3 | DTSFGGNLGR | 10 | A*68:01 | - |
| Peptide 4 | TSFGGNLGR | 9 | A*68:01 | - |
| Peptide 5 | GRAVFQAKK | 9 | B*27:05 | Donor # 475 |
| Peptide 6 | KRLLEPLGL | 9 | B*27:05 | - |
| Peptide 7 | QPAKKRLNF * | 9 | B*07:02 | - |
| Peptide 8 | RVITTSTRTW | 10 | B*57:01 | - |
| Peptide 9 | ITTSTRTW | 8 | B*57:01 | - |
| Peptide 10 | TRTWALPTY | 9 | B*27:05, C*07:01 | Donor # 505 |
| Peptide 11 | LPTYNNHLY | 9 | B*35:01, B*35:57 | Donor # 407 |
| Peptide 12 | KLFNIQVKEV | 10 | A*02:05, A*02:01 | - |
| Peptide 13 | TDSDYQLPYVL | 11 | B*40:02 ^ | Donor # 442 |
| Peptide 14 | DSDYQLPYVL | 10 | B*40:02 ^ | - |
| Peptide 15 | SDYQLPYVL | 9 | B*40:02 | - |
| Peptide 16 | IPQYGYLTL * | 9 | B*07:02 | Donor # 474, Donor # 519, Donor # 403, Donor # 513 |
| Peptide 17 | SQAVGRSSF * | 9 | B*15:01 | - |
| Peptide 18 | RSSFYCLEY | 9 | B*15:17 | - |
| Peptide 19 | LEYFPSQML | 9 | B*40:01, B*40:02 | Donor # 442 |
| Peptide 20 | LRTGNNFQF | 9 | B*27:05 | Donor # 403 |
| Peptide 21 | FQFSYEFENV | 10 | A*02:01 | Donor # 593, Donor # 399, Donor # 474, Donor # 519, Donor # 440, Donor # 403, Donor # 513 |
| Peptide 22 | SSYAHSQSL | 9 | C*03:04 | - |
| Peptide 23 | LMNPLIDQY | 9 | B*15:01 | - |
| Peptide 24 | LIDQYLYYL * | 9 | A*02:01 | - |
| Peptide 25 | GQNQQTLKF | 9 | B*15:01, C*07:01, C*02:02 | - |
| Peptide 26 | SVAGPSNMAVQGR | 13 | A*68:01 | Donor # 593, Donor # 475 |
| Peptide 27 | VAGPSNMAV | 9 | C*03:04 | - |
| Peptide28 | SEFAWPGASSW | 11 | B*44:02 | - |
| Peptide 29 | FAWPGASSW | 9 | B*57:01, B*35:01 | - |
| Peptide 30 | SLMNPGPAM | 9 | B*15:01 | - |
| Peptide 31 | RFFPLSGSLI | 10 | A*24:02 | - |
| Peptide 32 | RFFPLSGSLIF | 11 | A*23:01, A*24:02 | - |
| Peptide 33 | QSAQAQAQTGW | 11 | B*57:01 | - |
| Peptide 34 | LPGMVWQDRDVY | 12 | B*35:01 | - |
| Peptide 35 | MVWQDRDVY | 9 | B*35:01, B*35:57 | - |
| Peptide 36 | TPVPADPPTAF | 11 | B*35:01, B*35:57 | - |
| Peptide 37 | VPADPPTAF | 9 | B*07:02 | - |
| Peptide 38 | KLNSFITQY | 9 | A*03:01, B*15:01 | - |

| | | | | |
|---|---|---|---|---|
| Peptide 39 | KRWNPEIQY | 9 | B*27:05 | - |
| Peptide 40 | YYKSNNVEF | 9 | A*24:02, C*07:02 | - |
| Peptide 41 | YSEPRPIGTRY | 11 | A*01:01 | Donor # 325, Donor # 74 |

*- Previously reported immunodominant epitope. ^ - Low confidence allele prediction.

## Supplemental Table 6: Summary of peptide deconvolution for entire donor cohort

| Donor # | HLA-A alleles | HLA-B alleles | HLA-C alleles | Responding matrix pools (1 µg/mL) | Candidate peptides tested individually (2 µg/mL) | Previously reported epitopes tested individually (2 µg/mL) | Responding peptides |
|---|---|---|---|---|---|---|---|
| 74 | A*01:01<br>A*30:01 | B*08:01<br>B*42:01 | C*07:01<br>C*17:01G | MP 5, MP 13 | Peptide 41 | - | Peptide 41 |
| 202 | A*24:02<br>A*31:01 | B*35:01<br>B*39:05 | C*04:01<br>C*07:02 | - | - | - | - |
| 303 | A*23:01<br>A*30:04 | B*08:01<br>B*15:17 | C*03:04<br>C*07:01 | - | - | - | - |
| 325 | A*01:01<br>A*31:01 | B*08:01<br>B*39:11 | C*07:01<br>C*07:02 | MP 5, MP 7, MP 13 | Peptide 5, Peptide 41 | - | Peptide 41 |
| 345 | A*11:01<br>A*24:07 | B*15:21<br>B*35:05 | C*04:01<br>C*04:03 | MP 5 | - | - | - |
| 370 | A*01:01<br>A*31:01 | B*08:01<br>B*40:08 | C*03:04<br>C*07:01 | - | - | - | - |
| 371 | A*23:01<br>A*30:02 | B*18:01<br>B*53:01 | C*05:01<br>C*06:02 | MP 1, MP 3, MP 8, MP 10, MP 11, MP 13 | Peptide 7, Peptide 9, Peptide 19, Peptide 21, Peptide 25, Peptide 27, Peptide 37, Peptide 39 | - | - |
| 399 | A*02:05<br>A*24:02 | B*15:01<br>B*49:01 | C*01:02<br>C*07:01 | MP 3, MP 8, MP 10 | Peptide 9, Peptide 21 | Peptide 17 | Peptide 21 |
| 403 | A*02:01<br>A*30:01 | B*40:01<br>B*42:01 | C*03:04<br>C*17:01 | MP 1, MP 2, MP 3, MP 4, MP 7, MP 9, MP 10 | Peptides 1-4, Peptides 13-16, Peptides 19-22 | Peptide 24 | Peptide 16, Peptide 20, Peptide 21 |
| 406 | A*02:01<br>A*26:01 | B*14:01<br>B*35:01 | C*04:01<br>C*08:02 | MP 2, MP 5, MP 8, MP 12 | Peptide 8, Peptide 11, Peptide 32, Peptide 35 | Peptide 24 | - |
| 407 | A*02:02<br>A*03:01 | B*35:01<br>B*44:02 | C*04:01<br>C*05:01 | MP 4, MP 5, MP 8 | Peptide 10, Peptide 11 | - | Peptide 11 |
| 408 | A*01:01<br>A*02:01 | B*44:02<br>B*58:01 | C*05:01<br>C*07:18 | - | - | - | - |
| 440 | A*02:02<br>A*30:02 | B*07:02<br>B*15:10 | C*04:01<br>C*07:02 | MP 3, MP 10 | Peptide 21 | Peptide 7, Peptide 16 | Peptide 21 |
| 442 | A*03:01<br>A*32:01 | B*08:01<br>B*40:02 | C*02:02<br>C*07:01 | MP 1, MP 9, MP 10 | Peptide 13, Peptide 19 | - | Peptide 13, Peptide 19 |
| 474 | A*02:01<br>A*03:01 | B*07:02<br>B*44:02 | C*05:01<br>C*07:02 | MP 3, MP 4, MP 9, MP 10 | Peptide 15, Peptide 16, Peptide 21, Peptide 22 | Peptide 24 | Peptide 16, Peptide 21 |
| 475 | A*25:01<br>A*68:01 | B*27:05<br>B*44:02 | C*01:02<br>C*07:04 | MP 2, MP 3, MP 4, MP 5, MP 7, MP 8, MP 11 | Peptides 2-5, Peptides 8-11, Peptides 26-29 | - | Peptide 5, Peptide 26 |
| 505 | A*02:01<br>A*32:01 | B*27:05<br>B*44:02 | C*01:02<br>C*05:01 | MP 1, MP 3, MP 4, MP 6, MP 8, MP 10, MP 13 | Peptide 7, Peptide 9, Peptide 10, Peptide 12, Peptide 19, Peptide 21, Peptide 22, Peptide 24, Peptide 37, Peptide 39, Peptide 40 | - | Peptide 10 |
| 513 | A*02:01<br>~ | B*07:02<br>B*18:01 | C*07:02<br>C*07:04 | MP 1, MP 3, MP 4, MP 5, MP 9, MP 10, MP 11 | Peptide 13, Peptide 15, Peptide 16, Peptide 17, Peptide 19, Peptide 21, Peptide 22, Peptide 23, Peptide 25, Peptide 27, Peptide 28, Peptide 29 | Peptide 24 | Peptide 16, Peptide 21 |
| 514 | A*01:01<br>A*29:02 | B*44:03<br>B*57:01 | C*06:02<br>C*16:01 | - | - | - | - |
| 515 | A*03:01<br>A*33:03 | B*07:02<br>B*50:01 | C*06:02<br>C*07:02 | MP 4, MP 9 | Peptide 16 | Peptide 7 | - |

| | | | | | | | |
|---|---|---|---|---|---|---|---|
| 518 | A*03:01<br>A*24:02 | B*40:01<br>B*40:02 | C*02:02<br>C*03:04 | MP 1, MP 2, MP 3, MP 4, MP 7, MP 9, MP 10, MP 12, MP 13 | Peptides 1-4, Peptides 13-16, Peptides 19-22, Peptides 31-34, Peptides 37-40 | - | - |
| 519 | A*02:01<br>A*03:01 | B*07:02<br>B*13:02 | C*06:02<br>C*07:02 | MP 3, MP 10 | Peptide 21 | Peptide 7, Peptide 16, Peptide 24 | Peptide 16, Peptide 21 |
| 590 | A*01:01<br>A*03:01 | B*15:01<br>B*40:02 | C*02:02<br>C*03:03 | - | - | - | - |
| 593 | A*02:01<br>A*68:01 | B*07:02<br>B*44:02 | C*07:02<br>C*07:04 | MP 2, MP 3, MP 10, MP 11 | Peptide 20, Peptide 21, Peptide 26, Peptide 27 | Peptide 16, Peptide 24 | Peptide 21, Peptide 26 |

Predicted HLA binding alleles present in each donor are highlighted in red.

Note: Previously reported epitopes Peptide 16, Peptide 24, Peptide 7, and Peptide 17 were individually tested in multiple donors containing their respective HLA binding alleles even in the absence of response to the corresponding matrix pools.

**Supplemental Table 7: Comparison of measured binding affinities to predicted affinities**

| Peptide # | Epitope | Predicted binding allele(s) | Measured affinity (nM) (binding assay) | Predicted affinity (nM) (NetMHC) | % Rank (NetMHC) |
|---|---|---|---|---|---|
| Peptide 19 | LEYFPSQML | B*40:01 | 0.1 | 23.26 | 0.12 |
| Peptide 21 | FQFSYEFENV | A*02:01 | 0.6 | 8.82 | 0.09 |
| Peptide 41 | YSEPRPIGTRY | A*01:01 | 16.3 | 61.89 | 0.1 |
| Peptide 16 | IPQYGYLTL | B 07:02 | 31.1 | 17.83 | 0.09 |
| Peptide 5 | GRAVFQAKK | B*27:05 | 83.9 | 65.88 | 0.3 |
| Peptide 11 | LPTYNNHLY | B*35:01 | 96.7 | 8.24 | 0.04 |
| Peptide 10 | TRTWALPTY | B*27:05 | 120.2 | 589.67 | 1.6 |
| Peptide 20 | LRTGNNFQF | B 27:05 | 122.4 | 201.74 | 0.8 |
| Peptide 10 | TRTWALPTY | C*07:01 | >10^4 | 1287.83 | 0.5 |
| Peptide 19 | LEYFPSQML | B*40:02 | - | 138.29 | 0.5 |
| Peptide 13 | TDSDYQLPYVL | B 40:02 | - | 2699.35 | 3.5 |
| Peptide 11 | LPTYNNHLY | B*35:57 | - | - | - |
| Peptide 26 | SVAGPSNMAVQGR | A*68:01 | 1.9 | 835.02 | 3 |

Binding assay affinity cutoff for strong binders: < 100 nM. NetMHC % rank cutoff for strong binders: < 0.5.

**Supplemental Table 8: Clusters of HLA Class I MAPPs peptides**

| Cluster | Peptide # | Epitope | Peptide length | Predicted binding allele(s) | Donors with presentation on MAPPs assay (1) |
|---|---|---|---|---|---|
| 1 | Peptide 3 | DTSFGGNLGR | 10 | A*68:01 | Donor I |
| | Peptide 4 | TSFGGNLGR | 9 | A*68:01 | |
| 2 | Peptide 8 | RVITTSTRTW | 10 | B*57:01 | Donor I |
| | Peptide 9 | ITTSTRTW | 8 | B*57:01 | Donor J |
| 3 | Peptide 13 | TDSDYQLPYVL | 11 | B*40:02 ^ | Donor I |
| | Peptide 14 | DSDYQLPYVL | 10 | B*40:02 ^ | |
| | Peptide 15 | SDYQLPYVL | 9 | B*40:02 | |
| 4 | Peptide 26 | SVAGPSNMAVQGR | 13 | A*68:01 | Donor I |
| | Peptide 27 | VAGPSNMAV | 9 | C*03:04 | Donor J |
| 5 | Peptide 28 | SEFAWPGASSW | 11 | B*44:02 | Donors E, I |
| | Peptide 29 | FAWPGASSW | 9 | B*57:01, B*35:01 | Donors J, M |
| 6 | Peptide 31 | RFFPLSGSLI | 10 | A*24:02 | Donors A, F |
| | Peptide 32 | RFFPLSGSLIF | 11 | A*23:01, A*24:02 | Donor F |
| 7 | Peptide 34 | LPGMVWQDRDVY | 12 | B*35:01 | Donor M |
| | Peptide 35 | MVWQDRDVY | 9 | B*35:01, B*35:57 | Donors D, M, G |
| 8 | Peptide 36 | TPVPADPPTAF | 11 | B*35:01, B*35:57 | Donors D, M, G |
| | Peptide 37 | VPADPPTAF | 9 | B*07:02 | Donor A, F |

^- Low confidence allele prediction.

## REFERENCES

1. Brito-Sierra CA, Lannan MB, Malherbe LP, Siegel RW. The HLA class I immunopeptidomes of AAV capsid proteins. Front Immunol. 2023;14:1212136.
